# Use of Attention Deficit Hyperactivity Disorder Medication Among Danish Children and Adolescents From 2010 to 2023

**DOI:** 10.1111/acps.70103

**Published:** 2026-04-21

**Authors:** Maria Højgaard Stoltz‐Andersen, Martin Thomsen Ernst, Søren Dalsgaard, Lotte Rasmussen, Rikke Wesselhoeft

**Affiliations:** ^1^ Research Unit of Child and Adolescent Psychiatry Odense University Hospital Odense Denmark; ^2^ Clinical Pharmacology, Pharmacy and Environmental Medicine, Department of Public Health University of Southern Denmark Odense Denmark; ^3^ Child and Adolescent Mental Health Center, Copenhagen University Hospital – Bispebjerg and Frederiksberg Copenhagen Denmark; ^4^ Department of Clinical Medicine University of Copenhagen Copenhagen Denmark; ^5^ Department of Public Health, National Centre for Register‐Based Research Aarhus University Aarhus Denmark; ^6^ Research Unit of Child and Adolescent Psychiatry, Department of Clinical Research University of Southern Denmark Odense Denmark

**Keywords:** Denmark, drug utilization, pharmacoepidemiology, pharmacology, prescription rates, youths

## Abstract

**Introduction:**

Pharmacological treatment is an important component in the multimodal management of *attention deficit hyperactivity disorder* (ADHD), but contemporary trends in ADHD medication use among boys and girls in Denmark have not been fully characterized. This study aimed to provide an updated and detailed description of ADHD medication use among Danish children and adolescents during 2010–2023.

**Methods:**

We analyzed filled prescriptions for ADHD medication in youths aged 5–17 years in Denmark (2010–2023), calculating incidence rates, prevalence proportions, male/female ratios, treatment persistence, age at initiation, and prescriber setting. Analyses were stratified by sex and age.

**Results:**

The incidence rates of ADHD medication use followed a u‐shaped pattern for boys, declining from 0.59 per 100 person‐years in 2010 to 0.34 in 2014, before rising to 1.0 in 2023. Among girls, incidence rates increased continuously from 0.20 per 100 person‐years in 2010 to 0.67 per 100 person‐years in 2023. Prevalence proportions were stable until 2018, where an increase was observed for both sexes, reaching 3.4% for boys and 1.8% for girls in 2023. The male/female incidence and prevalence ratios decreased consistently from 2010 to 2023. ADHD medication was initiated at a median age of 13 years for girls and 11 years for boys. The share of index prescriptions from child and adolescent psychiatry increased, reaching 87% in 2023. Most 5–9‐year‐olds (65%) and almost half of 10–13‐year‐olds (45%) were covered by an ADHD medication prescription 5 years after treatment initiation, as opposed to 29% of 14–17‐year‐olds.

**Conclusion:**

ADHD medication use in Denmark increased from 2010 to 2023, particularly among girls, where the prevalence tripled compared to a 1.5‐fold increase in boys. This likely reflects greater awareness of ADHD in girls, although their 2‐year treatment delay compared to boys warrants clinical attention.

## Highlights

- Significant Outcomes
  ○ The use of ADHD medication among Danish children and adolescents increased markedly from 2010 to 2023, particularly among adolescent girls after 2019.
  ○ Girls initiated ADHD medication on average 2 years later than boys, but sex differences in prevalence diminished by late adolescence.
  ○ Most ADHD medication prescriptions were issued by doctors in child and adolescent psychiatry, indicating adherence to national clinical guidelines.
- Limitations
  ○ Findings may not generalize beyond the Danish healthcare system, which has universal coverage and specialist‐driven ADHD care.

## 1 Introduction

Attention deficit hyperactivity disorder (ADHD) affects 2.9%–8% of children and adolescents worldwide [1, 2], with a male‐to‐female ratio of approximately 2:1 [3, 4, 5]. The wide prevalence estimates may be due to a range of factors, including variations in diagnostic and treatment practices, cultural aspects, and study design. The sex disparity likely reflects differences in both biological and clinical factors [6, 7]. Girls more frequently exhibit the predominantly inattentive subtype of ADHD, which is less disruptive and more easily overlooked in structured settings [8, 9]. Moreover, psychiatric comorbidities co‐occurring with ADHD tend to differ by sex [10, 11]. These variations may contribute to sex‐specific differences in diagnostic recognition, help‐seeking behavior, and referral patterns, potentially leading to diagnostic and referral bias.

Sex‐specific variation is also evident in ADHD medication utilization, with studies consistently reporting higher usage among boys [5, 12, 13, 14, 15, 16]. A Finnish study found a male‐to‐female ratio of 4:1 among users [14], and recent Scandinavian data show that although boys consistently have higher prevalence of ADHD medication use than girls across all ADHD medication types, the sex difference narrows with increasing age [13]. Across the Nordic region, substantial between‐country variation exists, with Iceland showing the highest levels of ADHD medication use and Denmark and Finland generally exhibiting lower prevalence [17]. In Denmark, the sex gap has narrowed among 6–18‐year‐olds, reaching a male‐to‐female ratio of approximately 2:1 by 2022 [5]. In parallel, the prevalent use of ADHD medication has increased among Danish 6–18‐year‐olds since 1997, reaching 2.6% for girls and 5.3% for boys in 2022 [5]. While increased use may reflect improved recognition and access to treatment, concerns regarding potential overtreatment have also been raised.

Although ADHD is considered a long‐term condition, treatment is often discontinued [18]. A multinational study found that a higher proportion of Danish boys aged 4–11 years compared to girls remained on treatment 5 years after initiation, but no sex difference in long‐term treatment persistence was observed among adolescents [19]. This suggests a need for more detailed examination of utilization patterns among children and adolescents.

### 1.1 Aims of the Study

The aim of this study was to examine the use of ADHD medication among Danish children and adolescents from 2010 to 2023, thereby providing population‐level evidence on medication use that has not been updated in Denmark since 2012 [12, 20]. Using individual‐level data, we examined changes in the incidence rate and prevalence proportion of ADHD medication use over time, age at treatment initiation, treatment persistence, and the setting of prescribers issuing ADHD medication prescriptions. Due to sex‐specific disparities in ADHD prevalence between males and females, all analyses were stratified by both sex and age group.

## 2 Materials and Methods

### 2.1 Data Sources

Danish individuals aged 5–17 years who filled at least one prescription of ADHD medication during the years 2010–2023 were identified using the Danish National Prescription Registry. This registry contains information on all prescriptions filled at Danish community pharmacies since 1995 [21] and provides data on the dispensing date, prescriber identifier, quantity of dispensed medication (i.e., number of defined daily doses (DDD)), and the Anatomical Therapeutic Chemical (ATC) code defined by the World Health Organization (WHO) [22]. The register does not contain data on the intended duration of the single prescription, and we had no access to data on the indication for use. Information on vital status and migration was extracted from the Danish Civil Registration System, which is an administrative register covering all Danish residents since 1968 [23]. Total population counts were obtained from Statistics Denmark, which collects and stores electronic records for statistical and scientific purposes [24].

### 2.2 Study Population

We included all individuals living in Denmark who filled at least one prescription for ADHD medication at age 5–17 years in the period between January 1, 2010, and December 31, 2023. We distinguished between incident (new) and prevalent users. Incident users were defined as children and adolescents who filled their first‐ever prescription for an ADHD medication during the study period. Prevalent use was defined as the filling of at least one prescription. Individuals with a prior history of migration or who emigrated during the study period were excluded due to incomplete data.

### 2.3 ADHD Medication

We included methylphenidate (ATC code N06BA04), atomoxetine (N06BA09), guanfacine (C02AC02), dexamphetamine (N06BA02), and lisdexamphetamine (N06BA12), which are all indicated for treatment of ADHD in Denmark. Methylphenidate and atomoxetine were the only two medications available in the Danish market throughout the entire study period [25, 26]. Lisdexamphetamine was introduced to the Danish market in 2013 [27], and dexamphetamine and guanfacine in 2015 [28, 29].

### 2.4 Statistical Analyses

Descriptive analyses were used for the use of ADHD medication in children and adolescents from January 1, 2010, to December 31, 2023, and to characterize users. As the study focused on medication use rather than diagnostic status, analyses were based on dispensed ADHD medication and were not conditional on confirmed clinical diagnosis. Individuals were split into age groups of 5–9 years, 10–13 years, and 14–17 years based on age at the index prescription fill, and all analyses were stratified by sex and age group. We estimated the median age at the first‐ever ADHD prescription filled among incident users from January 1, 2010, to December 31, 2023. Medians were reported with interquartile ranges (IQR), and differences were examined using the Wilcoxon rank sum test, applying a 5% significance level. For both incidence rates and prevalence proportions, 95% confidence intervals were calculated assuming Poisson distributions.

#### 2.4.1 Incidence Rate of ADHD Medication Use

The annual incidence rate of ADHD medication use per 100 person‐years was calculated by dividing the number of new users each year by the total follow‐up time in person‐years among children and adolescents in the given year (corresponding to the total Danish population aged 5–17 years on January 1 the given year).

#### 2.4.2 The Prevalence of ADHD Medication Use

The annual prevalence proportion of ADHD medication users per 100 individuals was calculated by dividing the total number of prevalent users by the size of the total Danish population aged 5–17 years on January 1 the given year. The prevalence proportion was specified according to each ADHD medication and overall. Individuals could contribute to more than one medication group if they had filled prescriptions for multiple medications during the given year. Thus, the medication groups were not mutually exclusive.

#### 2.4.3 Treatment Persistence

Treatment persistence was estimated for incident users using a modified version of the Proportion of Patients Covered (PPC) method, which has been described in detail elsewhere [30]. In brief, this method allows individuals to have breaks in their treatment and hence estimates treatment persistence over multiple treatment episodes, as opposed to traditional treatment duration estimates (using Kaplan–Meier). Treatment initiation (Day 0) was defined as the date of the first recorded prescription fill for an ADHD medication, that is, the first‐ever prescription fill since 1995. The proportion of individuals covered by an ADHD prescription was estimated at fixed time points over a 5‐year period, assuming a single prescription provided drug coverage for 3 months. All individuals were followed for 5 years, even if they turned 18 years during follow‐up and could have transitioned from child and adolescent to adult psychiatric care. Censoring occurred upon death.

#### 2.4.4 Prescriber Setting

The clinical setting of the prescriber issuing each incident ADHD prescription was identified. Clinical settings were divided into general practice, child and adolescent psychiatry (secondary and primary healthcare), adult psychiatry (secondary and primary healthcare), pediatrics (secondary and primary healthcare), neurology (secondary and primary healthcare), and others. Prescriptions with missing prescriber information were excluded from the analysis. Individuals who had two prescriptions from two different prescribers on the same day were excluded.

All calculations and graphics were performed using STATA Release 18.0 (StataCorp, College Station, TX, USA).

### 2.5 Ethical Approval

The study was registered on the repository at the University of Southern Denmark (record no. 10.080). According to Danish legislation, purely register‐based studies do not need approval from an Ethics Committee [31].

## 3 Results

The study sample comprised 45,453 children and adolescents aged 5–17 years who filled an incident prescription for an ADHD medication in Denmark in 2010–2023. The majority were boys (68%), and the median age at first prescription was 11 years (IQR: 9–14). An additional 4774 individuals were excluded due to a history of migration.

### 3.1 Incidence Rates

**FIGURE 1:**
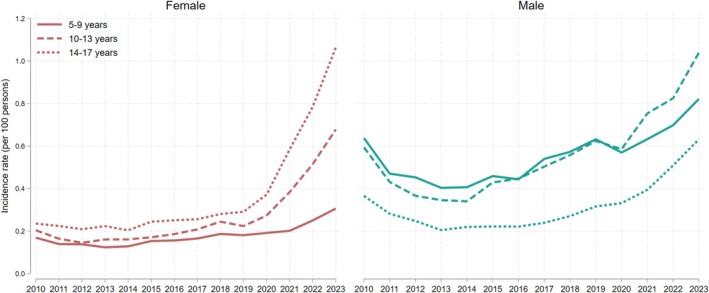
Annual incidence rates of ADHD medication use among Danish children and adolescents (5–17 years) from January 1, 2010 to December 31, 2023, stratified by sex and age at treatment initiation.

The overall incidence rates of ADHD medication use followed a u‐shape starting at 0.37 (95% CI 0.36–0.39) per 100 person‐years in 2010, dropping in 2013 to 0.25 (CI 0.24–0.26) and subsequently increasing to 0.75 (CI 0.73–0.77) in 2023 (Supporting Information S1). This pattern was observed for boys in all age categories (Figure 1, Supporting Information S1), most pronounced for 10–13‐year‐olds, where incident ADHD medication use per 100 person‐years almost halved from 0.59 (CI 0.55–0.64) in 2010 to 0.34 (CI 0.31–0.37) in 2014 and then increased to 1.0 (CI 0.99–1.1) in 2023 (Figure 1, Supporting Information S1). The incidence rates among girls were quite stable until 2019 and increased hereafter, most pronounced for 14–17‐year‐olds where they reached 1.1 (CI 1.0–1.1) per 100 person‐years in 2023 (Figure 1, Supporting Information S1).

The median age at first ADHD prescription was higher among girls (13 years, IQR: 10–16) than boys (11 years, IQR: 9–13) (*p* < 0.001) and was constant over time (data not shown).

### 3.2 Prevalence Proportions

**FIGURE 2:**
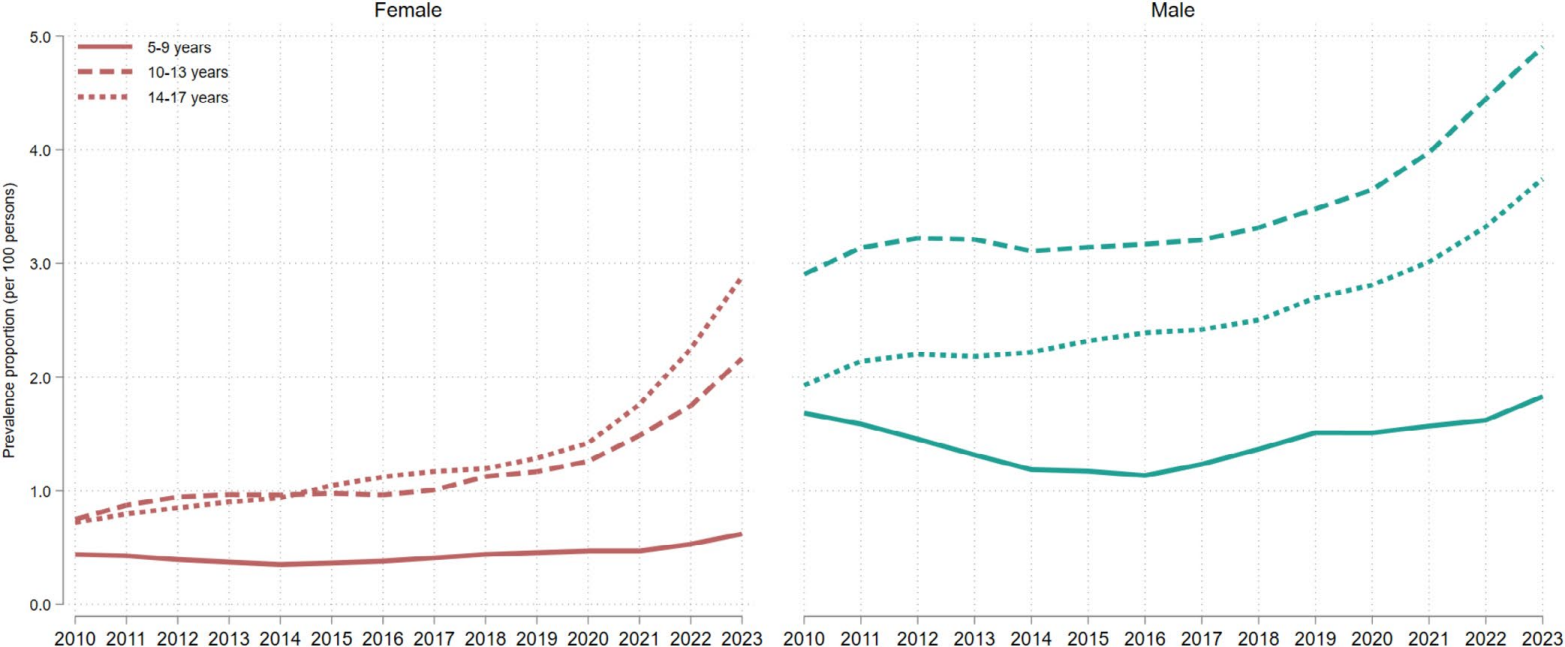
Annual prevalence proportions of ADHD medication use among Danish children and adolescents (5–17 years) from January 1, 2010 to December 31, 2023, stratified by sex and age group.

The prevalence of ADHD medication use increased steadily from 1.4% (CI 1.4–1.4) in 2010 to 2.6% (CI 2.6–2.7) in 2023, mainly due to greater use among 10–17‐year‐olds (Supporting Information S2). Prevalent use was higher for boys than girls throughout the study period (*p* < 0.001). Among boys, the highest prevalence was observed in 10–13‐year‐olds (4.9% in 2023) (Figure 2), which was significantly higher than for male age groups in the same year (*p* < 0.001) (Supporting Information S2). Among girls, the prevalence of ADHD medication use was increasingly higher for 10–17‐year‐olds than for 5–9‐year‐olds throughout the study period (Figure 2). However, prevalent use also increased among 14–17‐year‐olds from 2020 onwards, reaching 2.9% in 2023 (Supporting Information S2).

**FIGURE 3:**
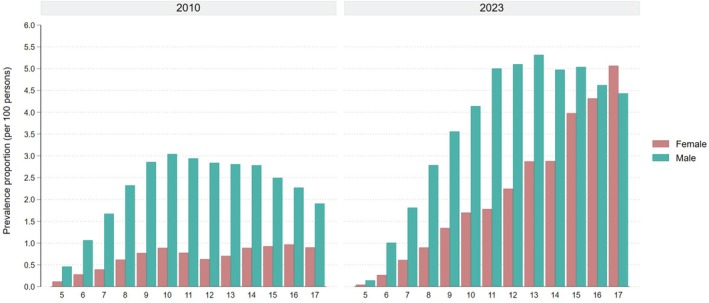
Prevalence proportions of ADHD medication stratified by age and sex in 2010 (left) and 2023 (right).

ADHD medication use peaked at age 17 years for girls, and at age 13 for boys in 2023 (Figure 3). ADHD medication use was predominantly used by preadolescents in 2010, but by 2023 this had changed to predominantly adolescent use. Furthermore, the sex differences in prevalent use had diminished in late adolescence in 2023, compared to 2010 (Figure 3).

**FIGURE 4:**
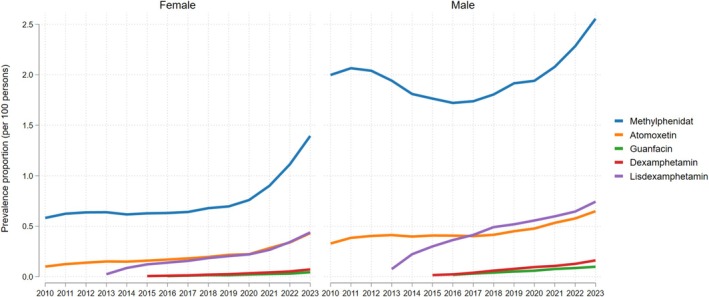
Annual prevalence of ADHD medication use among Danish children and adolescents (5–17 years) from January 1, 2010 to December 31, 2023, stratified by type of ADHD medication (an individual could use more than one medication in a given year).

Methylphenidate was the most commonly prescribed ADHD medication, regardless of age and sex (Figure 4). Prescription of atomoxetine increased steadily in the entire study period, from 0.33% (CI 0.31–0.35) to 0.65% (0.63–0.67) among boys, and from 0.10% (CI 0.09–0.11) to 0.43% (CI 0.41–0.45) among girls. Lisdexamphetamine was introduced in 2013, and prevalent use reached 0.74% (CI 0.72–0.77) among boys and 0.44% (CI 0.42–0.46) among girls in 2023 (Figure 4).

### 3.3 Treatment Persistence

**FIGURE 5:**
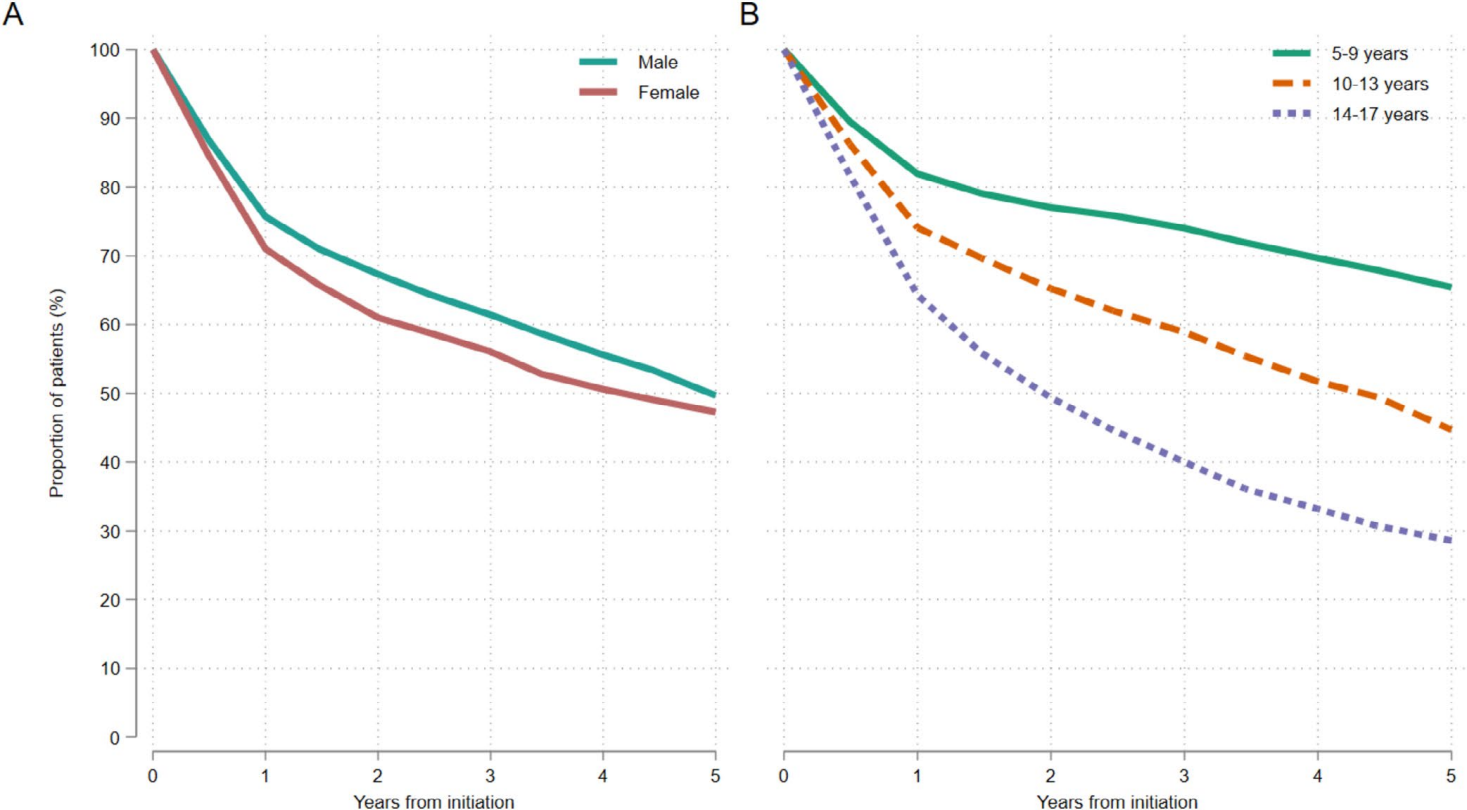
Treatment persistence (proportion of individuals covered by a prescription) among incident ADHD medication users from January 1, 2010 to December 31, 2023, stratified by (A) sex and (B) age at treatment initiation.

Children under 14 years old at ADHD medication initiation had higher treatment persistence than those initiating after age 13 (*p* < 0.001) (Figure 5, Supporting Information S3). Five years after the first prescription, 65% of 5–9‐year‐olds and 45% of 10–13‐year‐olds had an ADHD medication prescription, as opposed to 29% of those initiating treatment at 14–17 years (Figure 5, Supporting Information S3). Boys exhibited higher treatment persistence than girls throughout the 5‐year followup (50% vs. 47%, *p* < 0.001) (Supporting Information S3).

### 3.4 Prescriber Setting

Most index ADHD medication prescriptions were issued by doctors in child and adolescent psychiatry settings, ranging from 59% in 2010 and increasing to 87% in 2023. Index prescriptions from general practitioners decreased from 8.3% in 2010 to 0.33% in 2023 (Supporting Information S4). There were no differences in prescriber settings between boys and girls or between age groups (data not shown). A total of 13% of ADHD medication prescriptions were excluded due to missing prescriber information.

## 4 Discussion

We document an increase in use of ADHD medication among Danish children and adolescents from 2010 to 2023, especially during the last part of this period. There were distinct changes in utilization patterns over time, between boys and girls and between children and adolescents. Most notable was the initial decline in incident use of ADHD medication among boys, followed by a rise in the final years of the study period, while use among girls increased markedly from 2019 onwards. We also observed a shift towards predominantly adolescent use of ADHD medication in 2023 compared to preadolescent use in 2010. Age at treatment initiation was 2 years later for girls than for boys, although there was a catch‐up effect in late adolescence, where prevalent use almost leveled out between the sexes.

Our finding of a threefold increase in incidence rates of ADHD medication use among adolescent girls from 2010 to 2023 mirror results from Norway and Australia for the years 2006–2022 [32]. This reflects increased awareness of ADHD in girls [6, 9], as their symptoms have historically been overlooked or misattributed to anxiety or depressive disorders [6]. Improved recognition of sex‐specific presentations may therefore have contributed to increased identification and treatment in recent years. In contrast, the incidence rates of ADHD medication use among boys showed a clear U‐shaped pattern. The initial drop in incidence from 2010 to 2014 could reflect unwarranted skepticism expressed by the Danish Minister of Health [33] and increased societal awareness on the drawbacks of ADHD medication [34]. This may have caused youths, parents, and doctors to be reluctant about initiating pharmacological treatment, leading to a clearer reduction among males (who had a higher use than females at that time). The observed increase in incidence among boys from 2014 to 2019 could be partly due to the 2014 school reform that led to longer school days with additional hours dedicated to academic subjects [35]. These new demands may have been particularly challenging for boys with untreated ADHD, as ADHD in males is more often characterized by motor hyperactivity and behavioral symptoms that are more easily detected in school settings. In parallel with these educational changes, changes in national clinical guidance may have influenced prescribing patterns. The National Board of Health's Clinical Guideline on assessment and treatment of children and adolescents with ADHD [36] was published in 2014, presenting data on the low prevalence of ADHD medication use. Hence, it underlined that overprescribing of ADHD medications was unlikely and may have influenced the increase in incident ADHD medication use during 2014–2023.

The COVID‐19 pandemic coincided with an increased use of ADHD medication, and a 13% increase in Danish youths diagnosed with hyperkinetic disorders [37]. These results partly overlap with our findings of a prolonged rise in ADHD medication use until 2023. It is possible that school lockdowns, abrupt changes of family routines, and social isolation triggered children and adolescents, perhaps especially girls, with unrecognized ADHD to seek help [38]. Initiation of non‐pharmacological interventions could also be more difficult during the pandemic, leaving some patients with medication treatment choices only. The increasing use of ADHD medication over time, and especially post‐pandemic [39], calls for continuous and regular monitoring to facilitate rational use.

Although we document an increased incident use of ADHD medication among Danish children and adolescents, prevalent use was low and similar to that in Norway and Finland, and lower than prevalent use in Sweden and Iceland [13, 17]. The marked heterogeneity across Nordic countries underscores the need for dedicated pan‐Nordic comparative studies to better understand how structural and clinical factors influence ADHD medication use.

Our data do not allow us to conclude whether the increasing use of ADHD medication in Danish children and adolescents reflects rational use as opposed to under‐ or over‐prescribing. However, the observed proportion of prevalent users (2.6% in 2023) was much lower than the estimated worldwide prevalence of childhood ADHD [40, 41, 42]. It was also lower than the national prevalence of ADHD among Danish children aged 6–18, estimated at 5.3% for boys and 2.7% for girls in 2022 [5].

A notable shift in the age distribution of prevalent ADHD medication users was observed over the study period, from predominantly preadolescents in 2010 to adolescents in 2023. This may reflect increasing awareness of ADHD symptoms manifesting or persisting in adolescence. In parallel, treatment initiation was approximately 2 years later for girls than for boys. This is supported by a Swedish study showing a four‐year delay of ADHD diagnosis for females compared to males [43] and indicates that there is still an unmet potential for timely identification and treatment of young girls with ADHD in Denmark.

Almost all index ADHD medication prescriptions were issued by doctors working in child and adolescent psychiatry, which is in accordance with Danish clinical guidelines [44]. The use of specific ADHD medications also reflected guidelines for first‐ and second‐line treatments, with methylphenidate surpassing other ADHD medications [45].

The observation that 45%–65% of Danish children and 29% of adolescents were covered by an ADHD medication prescription 5 years after treatment initiation aligns with findings from other high‐income countries, where 50%–60% of children and 30%–40% of adolescents filled prescriptions after 5 years [19]. Danish children consistently exhibit higher treatment persistence relative to international peers [19], which could reflect higher treatment inertia and/or more targeted use of pharmacological treatment in Denmark. ADHD medication in Denmark is primarily prescribed by doctors within child and adolescent psychiatry, which may restrict medication to children with severe symptoms and a high need for continued pharmacological treatment. We found higher persistence with ADHD medication among children than adolescents, probably because of the greater reliance on parental support for compliance [46]. It is also possible that children with clearly recognizable ADHD symptoms in childhood have a higher treatment efficiency and/or tolerance than individuals diagnosed with ADHD in adolescence [47]. A recent systematic review found that younger age at diagnosis and specialist treatment were both associated with increased adherence and persistence with ADHD medication among children [18]. Sole authorization of index ADHD medication prescriptions by doctors within child and adolescent psychiatry could ensure appropriate dosing and enhance both monitoring and family support, thereby fostering better adherence and continuity of treatment.

A strength of our study is the use of a national prescription register with high completeness and validity [48], thus minimizing the risk of selection and recall bias. Our study also has some limitations. First, the exclusion of individuals with prior migration history may reduce the applicability of the findings to populations with migrant backgrounds, and the inability to align numerator and denominator with respect to migration history may have introduced a minor degree of imprecision in incidence and prevalence estimates. Second, our study relied on filled prescriptions as proxies for medication use, and it is unknown whether the medication was consumed. However, filled prescriptions are more indicative of medication intake than prescriptions alone [49] and are considered superior to self‐reported medication use. Third, our follow‐up period for adolescents could have covered a transition to adult psychiatric care, which may have affected long‐term persistence estimates due to different prescription practices. Fourth, we had no information on the intended duration of each dispensing nor the indication for use, and some medications may have been used off‐label for other conditions. Fifth, we did not present regional information on ADHD medication use, as recent studies have dealt with this in more detail, showing large differences in medication use across Denmark [50, 51].

## 5 Conclusions

We document an increase in the use of ADHD medication among children and adolescents in Denmark from 2010 to 2023. The pronounced sex differences in utilization patterns over time warrant further investigation into potential explanatory factors, including changes in diagnostic awareness for boys and girls, respectively. The increased ADHD medication use in the recent study years and the later treatment onset among girls call for continuous and regular monitoring to facilitate rational use and optimal treatment for both sexes. Reassuringly, ADHD medication was almost exclusively prescribed by doctors in child and adolescent psychiatry in accordance with Danish clinical guidelines. To further strengthen the interpretation of utilization patterns, future studies combining diagnostic and prescription data are needed to investigate indication‐specific use of ADHD medication.

## Author Contributions

R.W. and L.R. conceived the study idea. M.H.S.‐A., R.W., and L.R. designed the study and directed the analyses, which were carried out by M.T.E. M.H.S.‐A., R.W., and L.R. drafted the first manuscript. All authors participated in the discussion and interpretation of the results. All authors critically revised the manuscript and approved the final version of the article. All listed authors meet authorship criteria.

## Funding

The study was funded by a scholarship grant (to Maria Højgaard Stoltz‐Andersen) from the BRIDGE (Brain Research—Inter Disciplinary Guided Excellence) Foundation, University of Southern Denmark. Dr. Dalsgaard's research is currently supported by grants from the European Union's Horizon 2020 research and innovation program under grant agreements No 965381 (TIMESPAN), The Capital Region (grant No 22042850), and from Greater Copenhagen Health Science Partners (CAG Precision Psychiatry, A7763).

## Disclosure

All authors certify that they have no affiliations with or involvement in any organization or entity with any financial interest or non‐financial interest in the subject matter or materials discussed in this manuscript.

## Ethics Statement

The study was registered on the repository at the University of Southern Denmark (record no. 10.080). According to Danish legislation, purely register‐based studies do not need approval from an Ethics Committee.

## Conflicts of Interest

The authors declare no conflicts of interest.

## Supporting information


**Supporting Information: S1.** Incidence rates of ADHD medication use per 100 person‐years in Denmark from January 1, 2010, to December 31, 2023, reported with 95% confidence intervals.

**Supporting Information: S2.** Prevalence proportions of ADHD medication use per 100 individuals in Denmark from January 1, 2010, to December 31, 2023, reported with 95% confidence intervals and *p*‐values from test in differences between prevalence proportions.

**Supporting Information: S3.** Proportion of individuals covered by a prescription over a 5‐year period among incident users of ADHD medication from January 1, 2010, to December 31, 2023, stratified by sex and age at treatment initiation.

**Supporting Information: S4.** The annual proportions of prescribers authorizing the index ADHD medication prescriptions to Danish children and adolescents (5–17 years) according to clinical setting, from January 1, 2010, to December 31, 2023.

## Data Availability

Due to Danish legislation, individual‐level data are not available. Anonymized data can be made available for authorized researchers after application to Forskerservice at the Danish Health Data Authority.
