# Supplementary material for: Use of Attention Deficit Hyperactivity Disorder Medication Among Danish Children and Adolescents From 2010 to 2023
Source: Acta Psychiatr Scand. 2026 Apr 21;154(3):203–11. doi: 10.1111/acps.70103 (PMC13429349; doi:10.1111/acps.70103)
Supplement: Supplementary file 2 — Supporting Information: S2. Prevalence proportions of ADHD medication use per 100 individuals in Denmark from January 1, 2010, to December 31, 2023, reported with 95% confidence intervals and p‐values from test in differences between prevalence proportions. [file ACPS-154-203-s001.docx]

**Supplementary material 2** *Prevalence proportions of ADHD medication use per 100 individuals in Denmark from January 1, 2010, to December 31, 2023, reported with 95% confidence intervals and p-values from test in differences between prevalence proportions.*

|  | **2010**  **Prevalence (CI)** | **2011**  **Prevalence (CI)** | **2012**  **Prevalence (CI)** | **2013**  **Prevalence (CI)** | **2014**  **Prevalence (CI)** | **2015**  **Prevalence (CI)** | **2016**  **Prevalence (CI)** | **2017**  **Prevalence (CI)** | **2018**  **Prevalence (CI)** | **2019**  **Prevalence (CI)** | **2020**  **Prevalence (CI)** | **2021**  **Prevalence (CI)** | **2022**  **Prevalence (CI)** | **2023**  **Prevalence (CI)** |
| --- | --- | --- | --- | --- | --- | --- | --- | --- | --- | --- | --- | --- | --- | --- |
| **Total** | 1.4 (1.4-1.4) | 1.5 (1.5-1.5) | 1.5 (1.5-1.5) | 1.5 (1.4-1.5) | 1.4 (1.4-1.5) | 1.5 (1.4-1.5) | 1.5 (1.5-1.5) | 1.5 (1.5-1.6) | 1.6 (1.6-1.7) | 1.8 (1.7-1.8) | 1.8 (1.8-1.9) | 2.0 (2.0-2.0) | 2.3 (2.2-2.3) | 2.6 (2.6-2.7) |
| **Male** | 2.1 (2.1-2.2) | 2.2 (2.2-2.3) | 2.2 (2.2-2.3) | 2.2 (2.1-2.2) | 2.1 (2.1-2.1) | 2.1 (2.1-2.2) | 2.2 (2.1-2.2) | 2.2 (2.2-2.3) | 2.3 (2.3-2.4) | 2.5 (2.5-2.6) | 2.6 (2.6-2.7) | 2.8 (2.7-2.9) | 3.1 (3.0-3.1) | 3.4 (3.4-3.5) |
| **Female** | 0.63 (0.60-0.65) | 0.68 (0.66-0.71) | 0.71 (0.69-0.74) | 0.72 (0.70-0.75) | 0.73 (0.70-0.75) | 0.77 (0.74-0.80) | 0.79 (0.77-0.82) | 0.83 (0.81-0.86) | 0.89 (0.86-0.92) | 0.95 (0.92-0.98) | 1.0 (1.0-1.1) | 1.2 (1.2-1.3) | 1.5 (1.4-1.5) | 1.8 (1.8-1.9) |
| **Male-to-female ratio** | 3.4 | 3.3 | 3.1 | 3.0 | 2.9 | 2.8 | 2.7 | 2.7 | 2.6 | 2.7 | 2.5 | 2.31 | 2.1 | 1.9 |
| **5–9-year-olds** | | | | | | | | | | | | | | |
| **Male** | 1.7 (1.6-1.8) | 1.6 (1.5-1.7) | 1.5 (1.4-1.5) | 1.3 (1.3-1.4) | 1.2 (1.1-1.2) | 1.2 (1.1-1.2) | 1.1 (1.1-1.2) | 1.2 (1.2-1.3) | 1.4 (1.3-1.4) | 1.5 (1.5-1.6) | 1.5 (1.5-1.6) | 1.6 (1.5-1.6) | 1.6 (1.6-1.7) | 1.8 (1.8-1.9) |
| **Female** | 0.44 (0.41-0.47) | 0.43 (0.40-0.46) | 0.40 (0.37-0.43) | 0.37 (0.34-0.41) | 0.35 (0.33-0.38) | 0.37 (0.34-0.40) | 0.38 (0.35-0.41) | 0.41 (0.38-0.44) | 0.44 (0.41-0.48) | 0.46 (0.49-0.49) | 0.47 (0.44-0.51) | 0.47 (0.44-0.51) | 0.53 (0.50-0.57) | 0.62 (0.58-0.66) |
| **Male-to-female ratio** | 3.8 | 3.7 | 3.7 | 3.5 | 3.4 | 3.2 | 3.0 | 3.0 | 3.1 | 3.3 | 3.2 | 3.3 | 3.1 | 2.9 |
| **10-13-year-olds** | | | | | | | | | | | | | | |
| **Male** | 2.9 (2.8-3.0) | 3.1 (3.1-3.2) | 3.2 (3.1-3.3) | 3.2 (3.1-3.3) | 3.1 (3.0-3.2) | 3.1 (3.1-3.2) | 3.2 (3.1-3.3) | 3.2 (3.1-3.3) | 3.3 (3.3-3.4) | 3.5 (3.4-3.6) | 3.7 (3.6-3.8) | 4.0 (3.9-4.1) | 4.5 (4.3-4.6) | 4.9 (4.8-5.0) |
| **Female** | 0.75 (0.70-0.80) | 0.87 (0.83-0.93) | 0.95 (0.90-1.0) | 0.97 (0.92-1.0) | 0.97 (0.92-1.0) | 0.98 (0.93-1.0) | 0.97 (0.96-1.1) | 1.0 (1.0-1.2) | 1.1 (1.1-1.2) | 1.2 (1.2-1.3) | 1.3 (1.2-1.3) | 1.5 (1.4-1.5) | 1.8 (1.7-1.8) | 2.2 (2.1-2.2) |
| **Male-to-female ratio** | 3.9 | 3.6 | 3.4 | 3.3 | 3.2 | 3.2 | 3.3 | 3.2 | 2.9 | 3.0 | 2.9 | 2.7 | 2.5 | 2.3 |
| **14–17-year-olds** | | | | | | | | | | | | | | |
| **Male** | 1.9 (1.9-2.0) | 2.1 (2.1-2.2) | 2.2 (2.1-2.3) | 2.2 (2.1-2.3) | 2.2 (2.1-2.3) | 2.3 (2.2-2.4) | 2.4 (2.3-2.5) | 2.4 (2.3-2.5) | 2.5 (2.4-2.6) | 2.7 (2.6-2.8) | 2.8 (2.7-2.9) | 3.0 (2.9-3.1) | 3.3 (3.2-3.4) | 3.8 (3.7-3.9) |
| **Female** | 0.72 (0.68-0.77) | 0.80 (0.75-0.85) | 0.85 (0.80-0.90) | 0.90 (0.85-0.96) | 0.94 (0.89-0.99) | 1.1 (0.99-1.1) | 1.1 (1.1-1.2) | 1.2 (1.1-1.2) | 1.2 (1.2-1.3) | 1.3 (1.2-1.4) | 1.4 (1.4-1.5) | 1.8 (1.7-1.8) | 2.3 (2.2-2.3) | 2.9 (2.8-3.0) |
| **Male-to-female ratio** | 2.7 | 2.7 | 2.6 | 2.4 | 2.4 | 2.2 | 2.1 | 2.1 | 2.1 | 2.1 | 2.0 | 1.7 | 1.5 | 1.3 |
| **Test in differences between prevalence proportions (p-value)** | | | | | | | | | | | | | | |
| **Female vs. Male** | <0.001 | <0.001 | <0.001 | <0.001 | <0.001 | <0.001 | <0.001 | <0.001 | <0.001 | <0.001 | <0.001 | <0.001 | <0.001 | <0.001 |
| **5–9-year-olds vs. 10–13-year-olds** | <0.001 | <0.001 | <0.001 | <0.001 | <0.001 | <0.001 | <0.001 | <0.001 | <0.001 | <0.001 | <0.001 | <0.001 | <0.001 | <0.001 |
| **5–9-year-olds vs. 14-17-year-olds** | <0.001 | <0.001 | <0.001 | <0.001 | <0.001 | <0.001 | <0.001 | <0.001 | <0.001 | <0.001 | <0.001 | <0.001 | <0.001 | <0.001 |
| **10-13-year-olds vs. 14-17-year-olds** | <0.001 | <0.001 | <0.001 | <0.001 | <0.001 | <0.001 | <0.001 | <0.001 | <0.001 | <0.001 | <0.001 | <0.001 | <0.001 | <0.001 |

**Article title:** Use of attention deficit hyperactivity disorder medication among Danish children and adolescents from 2010-2023

**Journal name:** ACTA Psychiatrica Scandinavia

**Author names and affiliations:**

Maria Højgaard Stoltz-Andersen

Research Unit of Child and Adolescent Psychiatry, Mental Health Services in the Region of Southern Denmark, Odense, Denmark

Clinical Pharmacology, Pharmacy and Environmental Medicine, Department of Public Health, University of Southern Denmark, Odense, Denmark

Martin Thomsen Ernst

Clinical Pharmacology, Pharmacy and Environmental Medicine, Department of Public Health, University of Southern Denmark, Odense, Denmark

Søren Dalsgaard

Child and Adolescent Mental Health Center, Copenhagen University Hospital – Mental Health Services CPH, Copenhagen, Denmark

Department of Clinical Medicine, University of Copenhagen, Copenhagen, Denmark

National Centre for Register-based Research, Department of Public Health, Aarhus University, Aarhus, Denmark

Lotte Rasmussen

Clinical Pharmacology, Pharmacy and Environmental Medicine, Department of Public Health, University of Southern Denmark, Odense, Denmark

Rikke Wesselhoeft

Research Unit of Child and Adolescent Psychiatry, Department of Clinical Research, University of Southern Denmark

Research Unit of Child and Adolescent Psychiatry, Mental Health Services in the Region of Southern Denmark, Odense, Denmark

**E-mail address of the corresponding author**: [rwesselhoeft@health.sdu.dk](mailto:rwesselhoeft@health.sdu.dk)
